# Decision-making on preservation effort and inventory volume for fresh retailer under strategic consumer behavior

**DOI:** 10.1371/journal.pone.0341455

**Published:** 2026-03-20

**Authors:** Chuanbo Zhu, Whenyou Zhan

**Affiliations:** 1 Alibaba Business School, Hangzhou Normal University, Hangzhou, China; 2 College of Humanities and Arts, Jiaxing Nanhu University, Jiaxing, China; NOVA School of Science and Technology: Universidade Nova de Lisboa Faculdade de Ciencias e Tecnologia, PORTUGAL

## Abstract

Based on the theory of rational expectations equilibrium, this study examines the optimal decision-making of fresh retailer under strategic customer behavior. The paper focuses on analyzing the impact of perceived customer value on the retailer’s investment in freshness preservation and configuration in inventory, as well as how market size moderates this influence. The findings reveal that in the face of strategic customer behavior and an increased perception of value for the freshness of products nearing their sell-by date, the retailer tends to lower retail prices, which subsequently affects their enthusiasm for investment in freshness preservation efforts and inventory configuration. The influence of perceived customer value on preservation effort and inventory volume is differential and significantly moderated by market size. When the market size is large, the retailer is inclined to implement optimal preservation measures, but inventory volume may be negatively impacted. With a medium market size, the retailer reduces preservation efforts and inventory levels. However, in situations where perceived customer value is low, the retailer increases inventory volumes. When the market size is small, the impact of strategic customer behavior and perceived customer value on preservation efforts and inventory volumes more pronounced.

## 1 Introduction

Providing consumers with fresher agriculture products has become a competitive strategy for fresh retail businesses [1]. Statistics indicate that the annual distribution loss rate of fresh fruits and vegetables in China reaches 25%−35% [2]. Fresh produce is susceptible to spoilage during extended transportation periods. Indeed, in the absence of effective preservation methods, both the quantity and quality of the fresh goods can suffer significant deterioration [3–5]. As consumers’ awareness of health issues increases, freshness has become a significant factor influencing their preferences and willingness to pay for fresh agricultural products [5]. Due to the perishable nature of fresh goods, appropriate measures need to be taken during transportation and storage, such as using specialized refrigerated transport vehicles and cold storage facilities, to slow down the natural decay process and maintain or enhance the freshness of the products [6]. In fact, fresh agricultural products have a limited shelf life. Retailers often resort to markdowns for products nearing their expiration dates to minimize losses. However, the dynamic pricing of fresh products has led to more strategic behavior among consumers. Consumers, focusing on the freshness of products, are increasingly adopting strategic purchasing behaviors, guiding their buying decisions based on current prices, expectation of future price, and the availability of stock on hand [7,8]. Consumers’ expectations for inventory availability not only influences their timing of purchase decisions, but also their perception of the value of freshness for discounted products plays a similar role in affecting the timing of their choices. In this paper, we introduce the concept of perceived customer value and explain how a high perceived value of freshness for products on clearance sales is often detrimental to retailers. The perishable nature of fresh products and the presence of strategic consumer behavior pose certain challenges to business operations. How the retailer adjust their strategies of pricing, inventory volume and preservation effort in response to consumer strategic behavior is the focus of this paper.

The remainder of this paper is organized as follows. Sect 2 provides a literature review. In Sect 3, we formulate the basic model and analyse the optimal selling price, preservation effort and inventory volume. Sect 4 examines the retailer’s strategy under strategic customer behavior, as well as the impact of perceived customer value. Sect 5 analyzes and compares the direct impact of market size on retailers’ investment in freshness reservation and inventory configuration. Finally, a concluding remark is presented in Sect 6. All proofs are included in the Appendix.

## 2 Literature review

The literature related to this research is concentrated in two main areas: fresh produce preservation efforts and strategic customer behavior. The former primarily focuses on supply chain decision optimization for fresh produce, including pricing, order quantity/inventory, and preservation efforts; the latter mainly investigates the impact of strategic customer behavior on product pricing and inventory, as well as countermeasures.

### 2.1 Research on preservation efforts for fresh produce

Utilizing analytical models, many scholars investigate the issue from different aspects of decision making, such as inventory and replenishment [9–11], pricing [12,13], freshness preservation [4,14–16], joint ordering, pricing and cold chain logistics or freshness preservation [3,17–20], joint replenishment and freshness preservation [20–21], joint fresh preservation and other service [21–24]. Cai et al. [3] and Yu and Xiao [17] study the decision-making issues of pricing, order quantity, and preservation efforts for fresh produce. Babaee et al. [19] and Chen et al. [20] study the decisions of ordering, differentiated pricing, and preservation efforts for graded products. Dye and Hsieh [25] and Hsu et al. [26] construct an inventory model where the freshness of products varies over time and examined the retailer’s optimal replenishment strategy and level of preservation efforts. Liu and Hou [4] explore how to strategically plan cold chain logistics activities to achieve an appropriate balance among product freshness preservation, maximum delivery distance, and limited cold chain logistics budget. Chen et al. [14] study the retailer’s pricing and order quantity decisions and the supplier’s preservation efforts under demand information sharing, noting that retailers are willing to disclose demand information only when the freshness elasticity is high. some scholars explore the combined service strategies for fresh agricultural products. For instance, they have combined quality improvement efforts [22], advertising and promotional efforts [15], value-added services [22,23], carbon reduction and preservation efforts [22,27].

### 2.2 Research on strategic customer behavior

Su and Zhang [7] and Su [28] utilize the newsvendor model and the theory of rational expectations equilibrium to conduct a series of studies on habitual and speculative behaviors of strategic customers. They primarily investigate the optimal pricing and inventory strategies for retailers and examined the impact of strategic customer behavior on supply chain performance. Su [29] and Kremer et al. [30] explore dynamic pricing strategies for products under strategic customer behavior. Huang and Wang [31] study the impact of strategic customer behavior on pricing decisions of members in a remanufacturing supply chain. Wang et al. [32] focus on the pricing and inventory decisions of retailers under quick response strategies for risk-averse strategic consumers, analyzing the impact of such strategies on retailer profits. Chen et al. [33] discuss how to design a two-stage dynamic pricing strategy in the presence of both strategic customer behavior and reference price effects. Huang et al. [34] and He et al. [35] investigate the selection of retail channel sales models in the presence of strategic customer behavior. Najafi-Ghobadi et al. [8] explore dynamic pricing and advertising optimization strategies for two generations of products in the context of forward-looking customer behavior. The most relevant studies to this paper are those by Cai et al. [3] and Su and Zhang [7]. The former investigates pricing and freshness-keeping strategies for fresh produce, while the latter examines pricing and inventory strategies under strategic customer behavior. This paper introduces strategic customer behavior into the research on decision-making of pricing, inventory volume and preservation investment for fresh produce, and also considers the moderating effect of market size. For the first time, this paper studies the impact of strategic customer behavior and perceived customer value on the formulation of preservation effort and inventory for fresh agricultural produce. In the research by Su and Zhang [7], only strategic customer behavior was considered, without accounting for the impact of perceived customer value on retailer decision-making. Furthermore, this paper analyzes the influence of strategic customer behavior on retailers’ inventory and freshness preservation strategies, introducing market size as an important moderating variable and exploring its role in mitigating the adverse effects of such behavior on retailers.

## 3 Problem description and benchmark model

### 3.1 Problem description

Based on the newsvendor model, this paper explores the impact of strategic customer behavior on the decision-making of fresh produce retailers. Due to their perishable and seasonal nature, the demand for fresh agricultural products is significantly influenced by factors of freshness and randomness. To maintain the freshness of their products, retailers must implement a series of preservation measures. We define freshness as a function φ(τ) of the preservation efforts, where *τ* represents the level of preservation effort. Referring to Cai et al. [3], freshness is positively correlated with the level of preservation efforts and satisfies the following mathematical properties: $\frac{\partial \varphi (\tau )}{\partial \tau }>0,\;\frac{\partial^{2}\varphi (\tau )}{\partial \tau^{2}}<0,\;0\leq \varphi (\tau )\leq 1.$

We denote the freshness function as $\varphi (\tau )=\theta_{0}+\eta \theta (\tau )$, where $\theta_{0}$ represents the initial freshness level when the product reaches the consumer market without any preservation efforts, with $0\leq \theta_{0}\leq 1$. A higher $\theta_{0}$ indicates better inherent quality upon market arrival. Specifically, $\theta_{0}=0$ implies the product has no marketable freshness without preservation, while $\theta_{0}=1$ represents perfect freshness in the natural state. The term ηθ(τ) represents the additional freshness level achieved through preservation efforts, *η* represents the impact coefficient of preservation effort on the enhancement of freshness. Without loss of generality, we let $\theta_{0}=0$ and φ(τ)=ηθ(τ). To conveniently express the relationship between preservation efforts and freshness, we denote that $\varphi (\tau )=\eta \theta (\tau )=\eta \sqrt{\tau }$. Referring to HA [36], this paper adopts the following additive demand function D=λφ(τ)μ+ε to describe market demand. Here, *λ* is the market’s sensitivity to freshness, $\bar{\mu }$ is the average market size (hereinafter referred to as market size) and φ(τ)μ is the actual market size which is affected by freshness. *ε* is a random variable affecting market demand. To intuitively express random demand, similar to Huang et al. [34], we assume that *ε* follows the uniform distribution between *a* and *b*, i.e., ε~U(a,b) with probability density function f(·) and cumulative distribution function F(·).

The sale of fresh agricultural products is divided into two periods, i.e., the regular sales period and the salvage sales period. During the regular sales period, products are sold at the price *p* and cleared at the salvage value *s* during the salvage sales period. The retailer’s selling cost per unit of product is *c*. Without loss of generality, we suppose that s<c to prevent the retailer from profiting from the salvage value rather than normal sales [34,37]. Additionally, we assume that the cost of the retailer’s investment in preservation efforts is represented by a quadratic function $\frac{1}{2}k\tau^{2}$, where *k* is the preservation cost coefficient. Such a quadratic function has been widely adopted in the study of fresh produce preservation. It can well reflect the relationship between preservation efforts and costs, which has been confirmed by multiple studies [3,23,38]. Consistent with Su and Zhang [7] and Huang et al. [34], customers with strategic behavior may opt to purchase at full price during the regular sales period or choose to buy at a discount during the salvage sales period. Customers make purchase decisions based on a comparison of utilities between the two periods. Assuming that the utility customers derive from purchasing in the two periods is $U_{1}$ and $U_{2}$, respectively. Customers choose to purchase in the first period if $U_{1}>0$ and $U_{1}>U_{2}$, and purchase in the second period if $U_{2}>0$ and $U_{2}>U_{1}$. Assuming customers value products at *ν* during the normal sales period and at βν during the salvage sales period. Therefore, the utilities for customers purchasing in both periods are $U_{1}=\nu -p$ and $U_{2}=\xi (\beta \nu -s)$, respectively. Here, ξ represents the inventory availability, and *β* represents the perceived value of the product’s freshness during the salvage sales period (henceforth referred to as customer perceived value). Customers typically perceive a psychological reduction in value for fresh agricultural products approaching the end of their shelf life, which affects their purchasing intentions [39]. In Corollary 2, we will illustrate that if customers have a high perceived value for the freshness of products in the salvage sales period, it is actually detrimental to the retailer.

The decision sequence of this paper is as follows. Customers first establish a reservation price. Based on the expectation of reservation price, the retailer then determines the retail price, as well as the level of preservation effort and inventory. Consistent with Su and Zhang [7], customers have visibility only into the retail price, without access to information regarding the retailer’s inventory levels or the efforts made to preserve the product’s freshness. However, they can infer the availability of end-of-season sale products from the market demand distribution. Based on expectations of inventory availability, customers compare the utility of purchasing during the regular sales period versus the end-of-season sale period, and thus establish their reservation price *r* for purchasing during the regular sales period.

The condition for customers to choose to purchase during the regular sales period is $U_{1}>U_{2}$, i.e., ν−p≥ξ(βν−s). Hence, the customer’s reservation price is r=ν−ξ(βν−s). To avoid ambiguity, we assume that βν−s≥0. Although the retailer does not know the customer’s reservation price, they form an expectation of it based on the customer’s valuation of fresh produce, the perceived value of freshness, and the availability of inventory. Assuming the retailer’s expectation of the customer’s reservation price is $\xi_{r}$, then the retailer will set the price as $p=\xi_{r}$. We summarize the notation in Table 1.

**Table 1:** Notation.

Symbol	Description
*Parameters*	
*μ*	Market size.
*ε*	Random market demand.
f(·)	Probability density function of *ε*.
F(·)	Cumulative distribution function of *ε*.
*ν*	Customers’ valuation of fresh products during the regular selling period.
*β*	Customers’ perceived value of freshness during the residual-value (salvage) selling period.
*c*	Per-unit operating cost for the retailer.
*s*	Residual value of fresh products at market clearance.
ξ	Inventory availability during the residual-value selling period.
$U_{1}$	Utility from purchasing during the regular selling period.
$U_{2}$	Utility from purchasing during the residual-value selling period.
$\theta_{0}$	Initial freshness in the absence of preservation effort.
*λ*	Sensitivity of demand to freshness.
*η*	Impact coefficient of preservation effort on freshness.
$\xi_{r}$	Retailer’s anticipated reservation price of the consumer.
*Decisions*	
*r*	Customer reservation price.
*p*	Retail price.
*τ*	Preservation effort level invested by the retailer.
*Q*	Retailer’s inventory quantity.
*Functions*	
φ(τ)	Freshness of agricultural products.
*π*	Retailer’s profit function.
*Superscripts*	
*S*	Scenario with strategic consumer behavior.
*N*	Scenario without strategic consumer behavior.

**Definition 1.** *According to the theory of rational expectations equilibrium, there exists a rational expectations equilibrium that must satisfy the following conditions in the presence of strategic customer behavior:*

(i) r=ν−ξ(βν−s);
(ii) $p=\xi_{r}$;
(iii) $\left(Q,\tau )=\arg\max_{Q,\tau }\Pi (Q,p,\tau \right)$;
(iv) $\xi =F(Q-\lambda \varphi (\tau )\bar{\mu })$;
(v) $\xi_{r}=\xi$.

(i) illustrates the customer’s strategy of setting a reservation price for regular sales period purchases by weighing utilities across the two periods. (ii) and (iii) depict the retailer’s anticipation of the customer’s reservation price, which then informs the setting of the retail price during the regular sales period. Subsequently, the retailer determines the optimal preservation effort and inventory volume. (iv) and (v) establish a rational expectations equilibrium, mirroring the mutual expectations between the customer and the retailer. Specifically, (iv) captures the customer’s anticipation of inventory availability, whereas (v) captures the retailer’s anticipation of the customer’s reservation price.

### 3.2 Benchmark model

In the benchmark scenario, where it is assumed that all customers opt to purchase during the regular sales period, the retailer’s expected profit function is as follows:


$$\Pi_{N}(Q_{N},p_{N},\tau_{N})=p_{N}𝔼\min(Q_{N},D)+s\;𝔼\max(Q_{N}-D,0)-cQ_{N}-\frac{1}{2}k\tau_{N}^{2}.$$ (1)

This equation represents the retailer’s profit, where the retail price $p_{N}$, inventory volume $Q_{N}$, and preservation effort $\tau_{N}$ are decision variables. The first component on the right-hand side signifies the revenue from sales; the second component is the salvage value of unsold inventory; the third component encompasses operational expenses; and the last component accounts for the costs associated with preservation efforts.

We can rewrite the above equation in the following form:


$$\Pi_{N}(Q_{N},p_{N},\tau_{N})=(p_{N}-c)Q_{N}-(p_{N}-s)\int_{0}^{e^{Q_{N}-\lambda \varphi (\tau_{N})\bar{\mu }}}F(X)\;dX-\frac{1}{2}k\tau_{N}^{2}.$$ (2)

**Theorem 1.** *In the absence of strategic customer behavior, the retailer’s optimal decisions are as follows:*


$$(i)\;p_{N}^{*}=\nu$$ (3)

$$(\text{ii})\;\tau_{N}^{*}=\min\left\{\eta^{-2},{\left[\frac{\lambda \eta \bar{\mu }(\nu -c)}{2k}\right]}^{3/2}\right\};$$ (4)

$$(\text{iii})\;Q_{N}^{*}=\min\left\{\lambda \bar{\mu }+y_{N},\;(\lambda \eta \bar{\mu }{)}^{7/4}{\left[\frac{\left(\nu -c\right)}{2k}\right]}^{3/4}+y_{N}\right\}.$$ (5)

Here,


$$y_{N}=a+\frac{\left(b-a)(\nu -c\right)}{\left(\nu -s\right)}.$$

Theorem 1 illustrates the retailer’s optimal decision-making under the benchmark scenario. The retailer prices the product in line with its customer-assessed value. Following this, the retailer determines the optimal levels of preservation effort and sets the inventory volume.

## 4 The Newsvendor model with strategic consumer behavior

In this section, we examine the impact of strategic customer behavior on the retailer’s decision-making. In the context of strategic consumer behavior, customers strategically determine the timing of their purchases by weighing the utility of two purchasing periods. To encourage early purchases, the retailer, under rational expectations equilibrium, will set the optimal price at the level of the customer’s reservation price, that is $p^{*}=r^{*}=\nu -\xi (\beta \nu -s),$ where $\xi =F(Q-\lambda \varphi (\tau )\bar{\mu })$. The retailer’s profit function is shown as follows:


$$\Pi (Q,p,\tau )=p\;𝔼\min(Q,D)+s\;𝔼\max(Q-D,0)-cQ-\frac{1}{2}k\tau^{2}.$$ (6)

It can be observed that this profit function is similar to the one in the benchmark scenario, with the key difference lying in the retailer’s pricing strategy. In the benchmark scenario, the retailer sets the optimal price as $p_{N}^{*}=\nu$, whereas under strategic customer behavior, the retailer sets the optimal price as $p^{*}=\nu -\xi (\beta \nu -s)$.

**Theorem 2.** *Under strategic customer behavior, the optimal retail price and the impact of perceived value on the optimal retail price are as follows:*


$$\begin{array}{c} p^{*}=\left\{ \begin{array}{cc} \nu , & \beta =\frac{s}{\nu },\\ s+(1-\beta )\frac{\nu }{2}+\sqrt{\frac{(1-\beta {)}^{2}\nu^{2}}{4}+\beta \nu (c-s)+s^{2}-cs}, & \frac{s}{\nu }<\beta <1,\\ s+\sqrt{\left(\nu -s)(c-s\right)}, & \beta =1. \end{array}\right.\; \end{array}$$ (7)

Theorem 2 uncovers the optimal pricing strategy of the retailer under strategic customer behavior (hereafter referred to as SS). Specifically, the retailer’s optimal pricing is influenced by inventory availability and the perceived value of customers. It is important to note that when the perceived value *β* equals s/ν, customers choose to purchase during the regular sales period. However, the retailer sets the price identical to that in the benchmark scenario, which is akin to the benchmark situation. When the perceived value equals *1*, meaning customers perceive no change in the value of products between the two periods, the retailer sets the lowest possible price $p^{*}=s+\sqrt{\left(\nu -s)(c-s\right)}$, which is consistent with the findings of Su and Zhang [7].

**Corollary 1.** *Under strategic customer behavior, the sensitivity of the optimal retail price to perceived customer value is as follows:*


$$\frac{\partial p^{*}}{\partial \beta }<0\;\text{if}\;\beta \in \left(\frac{s}{\nu },1\right]$$

**Fig 1:**
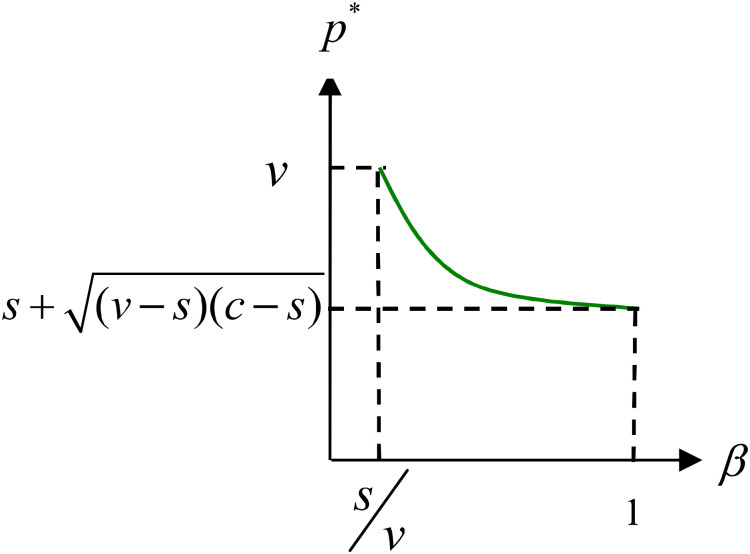
The impact of customer perceived value on retail price.

In response to an increase in perceived customer value, the retailer opts to lower their prices—an intriguing phenomenon. As customer-perceived value increases, the retailer is compelled to lower the retail price, a strategy that can be counterproductive. This reduction in pricing, as depicted in Fig 1, leads to a decrease in the retailer’s sales revenue, which is not in their best interest. Therefore, the retailer would prefer that customers maintain a lower perception of freshness for products during the end-of-season sales period.

**Theorem 3.** *Under strategic consumer behavior, the optimal levels of preservation effort and inventory volume are as follows:*


$$(\text{i})\;\tau_{S}^{*}=\min\left\{\eta^{-2},{\left[\frac{\lambda \eta \bar{\mu }(p^{*}-c)}{2k}\right]}^{3/2}\right\};$$ (8)

$$(\text{ii})\;Q_{S}^{*}=\min\left\{\lambda \bar{\mu }+y_{S},(\lambda \eta \bar{\mu }{)}^{7/4}{\left(\frac{\left(p^{*}-c\right)}{2k}\right)}^{3/4}+y_{S}\right\}.$$ (9)

Where


$$p^{*}=s+\frac{(1-\beta )\nu }{2}+\sqrt{\frac{(1-\beta {)}^{2}\nu^{2}}{4}+\beta \nu (c-s)+s^{2}-cs},\;\beta \in \left(\frac{s}{\nu },1\right],$$

and


$$y_{S}=a+\frac{p^{*}-c}{\nu -s}(b-a).$$

Theorem 3 further elucidates that the existence of strategic customer behavior incites retailers to lean towards diminishing their investment in product preservation and scaling back inventory commitments. The main driving force behind this inclination is the heightened customer-perceived value, which pressures the retailer to reduce the retail price, consequently dampening their enthusiasm for preservation and inventory planning.

**Corollary 2.** *Under strategic consumer behavior, the optimal levels of preservation effort and inventory volume are increased with* *η* *and decreased with* *k*.

(i) $\frac{\partial \tau_{S}^{*}}{\partial \eta }>0,\;\frac{\partial Q_{S}^{*}}{\partial \eta }>0;$
(ii) $\frac{\partial \tau_{S}^{*}}{\partial k}<0,\;\frac{\partial Q_{S}^{*}}{\partial k}<0.$

Corollary 2 explains the sensitivity of the optimal preservation effort and inventory volume to the coefficients of freshness enhancement and preservation cost. A higher coefficient of freshness enhancement and a lower coefficient of preservation cost indicate greater efficiency in preservation investment. Increased efficiency in preservation investment motivates the retailer to invest more in preservation efforts and to allocate larger inventories, which aligns with real-world practices.

**Corollary 3.** *Under varying market sizes, the sensitivity of the retailer’s optimal levels of preservation effort and inventory volume to perceived customer value are as follows:*

(i) $\frac{\partial \tau_{S}^{*}}{\partial \beta }=0,\;\text{if}\;\bar{\mu }\geq \mu_{S};$
(ii) $\frac{\partial \tau_{S}^{*}}{\partial \beta }<0,\;\text{if}\;\bar{\mu }<\mu_{S};$
(iii) $\frac{\partial Q_{S}^{*}}{\partial \beta }<0.$

Where, $\mu_{S}=2\lambda^{-1}\eta^{-7/3}k(p^{*}-c{)}^{-1}.$

Corollary 3 illustrates how an increase in customer-perceived value can adversely affect the retailer’s decision-making. According to Theorem 3, we observe that in larger market sizes, the levels of preservation effort reach their maximum value $\left(\eta^{-2}\right)$, at which point the retailer becomes less responsive to further increases in perceived customer value. However, in markets of moderate or smaller size, there is a positive correlation between preservation effort and retail price, and a negative correlation between retail price and customer-perceived value. This implies that if the market size is small or medium, perceived customer value has a negative impact on preservation efforts; whereas if the market size is large, it exerts no influence whatsoever. Nevertheless, regardless of market size, an increase in perceived value impacts the retailer’s enthusiasm for inventory allocation.

## 5 The moderating role of market size: Analytical and numerical results

Strategic customer behavior influences the retailer’s approach, but an expansion of market size can also mitigate this adverse impact. In this section, we conduct a comparative analysis of the direct impact of market size on retailer decisions under the two scenarios. The preceding theoretical propositions reveal how market size moderates the impact of strategic consumer behavior. To empirically ground these findings and quantify their managerial implications, we introduce numerical examples throughout this section. These analyses complement our theoretical results and provide actionable insights.

**Corollary 4.** *A comparison of the impact of market size on the optimal level of preservation effort in both scenarios.*

(i) $\tau_{N}^{*}\geq \tau_{S}^{*};$
(ii) $\frac{\partial \tau_{S}^{*}}{\partial \bar{\mu }}<\frac{\partial \tau_{N}^{*}}{\partial \bar{\mu }},\;\text{if}\;\bar{\mu }<\mu_{N};\;\frac{\partial \tau_{S}^{*}}{\partial \bar{\mu }}>\frac{\partial \tau_{N}^{*}}{\partial \bar{\mu }}=0,\;\text{if}\;\mu_{N}\leq \bar{\mu }<\mu_{S};$
(iii) $\frac{\partial \tau_{S}^{*}}{\partial \bar{\mu }}=\frac{\partial \tau_{N}^{*}}{\partial \bar{\mu }}=0,\;\text{if}\;\bar{\mu }\geq \mu_{S}.$

Where, $\mu_{N}=2\lambda^{-1}\eta^{-7/3}k(\nu -c{)}^{-1},\;\mu_{S}=2\lambda^{-1}\eta^{-7/3}k(p^{*}-c{)}^{-1}.$

**Fig 2:**
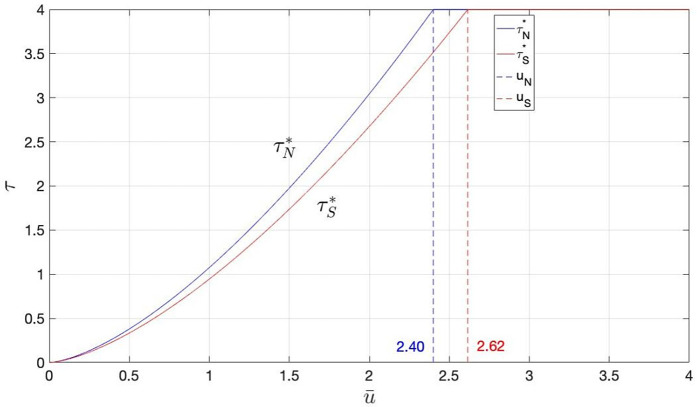
The impact of market size on the optimal preservation effort in two scenarios.

As a numerical illustration of Corollary 4, Fig 2 depicts the varying effects of market size on the retailer’s motivation to increase preservation efforts in both scenarios. The parameter values used in this example (λ=0.3,k=0.5,η=0.5,β=0.4,s=2,c=3,ν=10,a=0,b=1) are selected to adhere to the model’s fundamental assumptions (e.g., ν>c>s) and are consistent with scales used in related literature. Broadly speaking, a larger market size encourages the retailer to invest more in preservation, with those in the BS scenario always dedicating greater resources to preservation.

As shown in Fig 2, when the market is small $\left(\bar{\mu }<\mu_{N}\right)$, the size of the market has a more noticeable effect on enhancing preservation in the BS scenario. At a moderate market size $\left(\mu_{N}\leq \bar{\mu }<\mu_{S}\right)$, the market size more strongly influences preservation in the SS scenario, as the BS scenario’s preservation level has already hit its cap. In large markets $\left(\bar{\mu }\geq \mu_{S}\right)$, the preservation levels for both scenarios have reached their maximum, rendering market size ineffective in swaying the retailer’s preservation strategy.

**Fig 3:**
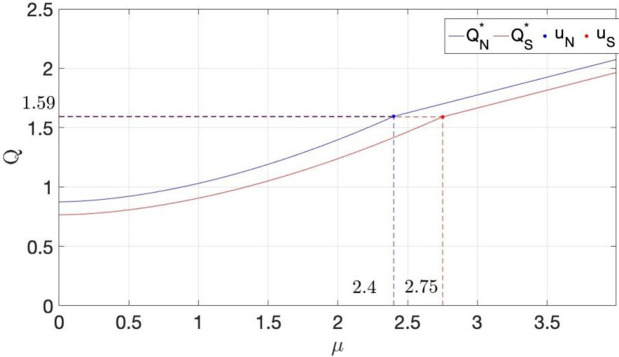
The impact of market size on the optimal inventory volume in two scenarios.

**Corollary 5.** *The impacts of market size on the optimal inventory in both scenarios are as follows:*

(i) $Q_{N}^{*}>Q_{S}^{*};$
(ii) $\frac{\partial Q_{S}^{*}}{\partial \bar{\mu }}<\frac{\partial Q_{N}^{*}}{\partial \bar{\mu }},\;\text{if}\;\bar{\mu }<\mu_{N};$
(iii) $\left\{ \begin{array}{cc} \frac{\partial Q_{S}^{*}}{\partial \bar{\mu }}\leq \frac{\partial Q_{N}^{*}}{\partial \bar{\mu }}=\lambda , & \text{when}\;p^{*}(\beta )\leq \bar{p},\\ \frac{\partial Q_{S}^{*}}{\partial \bar{\mu }}>\frac{\partial Q_{N}^{*}}{\partial \bar{\mu }}=\lambda , & \text{when}\;p^{*}(\beta )>\bar{p}, \end{array}\right.\;\text{if}\;\mu_{N}\leq \bar{\mu }<\mu_{S};$
(iv) $\frac{\partial Q_{S}^{*}}{\partial \bar{\mu }}=\frac{\partial Q_{N}^{*}}{\partial \bar{\mu }}=\lambda ,\;\text{if}\;\bar{\mu }\geq \mu .$ Where,

We further exemplify Corollary 5 through a numerical study, as shown in Fig 3, which illustrates the differential impact of market size on inventory configuration under the two scenarios. Similar to the conclusion of Corollary 4, the expansion of market size can significantly motivate retailers to increase their inventory levels, and under the BS scenario, retailers tend to maintain a higher inventory level. When the market size is small, the promotional effect of market size on the BS scenario is more pronounced. When the market size is medium, the differential promotional effect of market size on the two scenarios depends on the retail price. When the market size is large, the promotional effect of market size on both scenarios is consistent and entirely depends on the sensitivity coefficient of demand to freshness.

Combining Theorem 1 and 3, when the market size is moderate, the Benchmark Scenario (BS) reaches optimal freshness first, and the impact of market size on optimal inventory levels depends solely on the sensitivity coefficient of demand to freshness, independent of the retail price. At this time, in the Strategic Customer Behavior Scenario (SS), the products has not yet achieved optimal freshness, and the incentive effect of market size on inventory levels is still influenced by the retail price. Therefore, when the market size is moderate, the higher the retail price, the more pronounced the incentive effect of market size on inventory volume. Since the retail price is influenced by customer-perceived value, that is, if customers perceive a lower value, a higher retail price set by the retailer is beneficial for encouraging greater inventory allocation.

**Fig 4:**
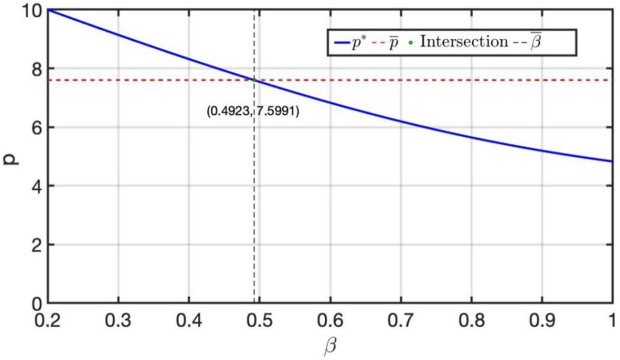
The intersection of $p^{*}(\beta )$ and $\bar{p}$.

As shown in Fig 4, the intersection point reveals critical thresholds for managerial decision-making. Specifically, $\bar{\beta }=0.49$ represents the perceived value threshold: when customers‘ perceived value *β* falls below 0.49, the retailer can set a higher price $p>\bar{p}$ (where $\bar{p}=7.60$ is the price threshold). The condition $p^{*}(\beta )>7.60)$ indicates that when the optimal price exceeds this threshold, market size expansion has a more pronounced incentive effect on inventory volumes in the Strategic Scenario compared to the Benchmark Scenario.

Combining Corollaries 4 and 5, we observe that an increase in market size has a positive effect in mitigating strategic customer behavior. In situations with a large market size, compared to Scenario BS, the retailer tends to elevate their preservation efforts to the highest level under Scenario SS, although the levels of inventory volume may still be subject to some influence. In contrast, in environments with a small market size, the impact of strategic customer behavior on preservation efforts and inventory configuration is more pronounced. At moderate market sizes, this impact is somewhat mitigated. A decrease in customer-perceived value helps to reduce the negative impact of strategic behavior on optimal inventory volumes. Overall, a larger market size helps retailers maintain higher levels of preservation effort. In the context of a smaller market size, the retailer may adopt strategies to reduce the levels of preservation effort and inventory volume to counteract strategic customer behavior. Therefore, even in the face of strategic consumer behavior, an expanded market size can alleviate its negative effects to a certain extent. In the real business environment, large retailers such as Amazon, Walmart, and JD.com, due to their market size advantage, are typically able to more effectively manage strategic customer behavior, maintaining higher levels of preservation investment and stable inventory configuration.

## 6 Conclusion

This study develops a theoretical model grounded in the rational expectations equilibrium framework to investigate the operational decisions of a fresh produce retailer confronting strategic consumer behavior. Our analysis is complemented by integrated numerical studies that visually elucidate the theoretical relationships and quantify their managerial implications. The findings reveal that confronted with strategic customer behavior and a high perceived value of product freshness during the market clearance period, the retailer is compelled to adopt price reduction strategies. The influence of perceived customer value on the retailer’s preservation effort and inventory volume is differential and significantly moderated by market size. In large-scale markets, the preservation effort tends to be maximized, while both preservation effort and inventory configuration are more adversely affected in small-scale markets. A medium-scale market, however, exhibits a certain buffering effect against these impacts.

These findings provide clear strategic guidance for retailers of different scales. For market leaders, their scale advantage allows them to entrench high-level preservation investment as a core competitive barrier. For medium-sized competitors, strategic flexibility is key, requiring dynamic adjustment of preservation and inventory strategies based on localized consumer perceptions. As for small-scale operators, the strategic focus should shift to rigorous inventory risk control and light-asset operations, significantly reducing capital expenditures while building advantages through community trust and personalized services.

Despite these contributions, this study is subject to several limitations that also define the boundaries of its applicability. The model primarily applies to perishable goods where freshness deterioration is a critical concern and strategic consumer behavior is pronounced, such as fresh agricultural products, flowers, and certain dairy items. Its insights are less directly transferable to non-perishable goods or services. Furthermore, our model operates under the assumptions of a homogeneous consumer population and a single-retailer setting. Future research could therefore explore the effects of consumer heterogeneity, competitive environments, and more complex multi-echelon supply chain structures to provide even more nuanced guidance for managers.

## Appendix: Proofs

***Proof of theorem 1.*** Substituting $p_{N}^{*}=\nu$ into Eq (2) and taking the first-order derivative with respect to $Q_{N}$ and $\tau_{N}$, respectively, yields


$$\frac{\partial \Pi_{N}}{\partial Q_{N}}=(\nu -c)-(\nu -s)F(Q_{N}-\lambda \varphi (\tau_{N})\bar{\mu }),$$

$$\frac{\partial \Pi_{N}}{\partial \tau_{N}}=-\frac{\lambda \bar{\mu }(\nu -s)}{b-a}\left[\frac{\partial \varphi (\tau_{N})}{\partial \tau_{N}}(a-Q_{N}+\lambda \varphi (\tau_{N})\bar{\mu })\right]-k\tau_{N}.$$

Taking the second-order derivatives of Eq (2) with respect to $Q_{N}$ and $\tau_{N}$, respectively, results in


$$\frac{\partial^{2}\Pi_{N}}{\partial Q_{N}^{2}}=-\frac{\left(\nu -s\right)}{b-a},$$

$$\frac{\partial^{2}\Pi_{N}}{\partial \tau_{N}^{2}}=-\frac{\lambda \bar{\mu }(\nu -s)}{b-a}\left[\frac{\partial^{2}\varphi (\tau_{N})}{\partial \tau_{N}^{2}}(a-Q_{N}+\lambda \varphi (\tau_{N})\bar{\mu })+\lambda \bar{\mu }{\left(\frac{\partial \varphi (\tau_{N})}{\partial \tau_{N}}\right)}^{2}\right]-k,$$

$$\frac{\partial^{2}\Pi_{N}}{\partial Q_{N}\partial \tau_{N}}=\frac{\lambda \bar{\mu }(\nu -s)}{b-a}\frac{\partial \varphi (\tau_{N})}{\partial \tau_{N}},\;\frac{\partial^{2}\Pi_{N}}{\partial \tau_{N}\partial Q_{N}}=\frac{\lambda \bar{\mu }(\nu -s)}{b-a}\frac{\partial \varphi (\tau_{N})}{\partial \tau_{N}}.$$

Calculating the Hessian matrix gives


$$\begin{array}{c} H=\frac{\nu -s}{b-a}[\begin{array}{cc} -1 & \lambda \bar{\mu }\frac{\partial \varphi (\tau_{N})}{\partial \tau_{N}}\\ \lambda \bar{\mu }\frac{\partial \varphi (\tau_{N})}{\partial \tau_{N}} & -\lambda \bar{\mu }\left[\frac{\partial^{2}\varphi (\tau_{N})}{\partial \tau_{N}^{2}}(a-Q_{N}+\lambda \varphi (\tau_{N})\bar{\mu })+\lambda \bar{\mu }{\left(\frac{\partial \varphi (\tau_{N})}{\partial \tau_{N}}\right)}^{2}\right]-k\frac{b-a}{\nu -s} \end{array}].\; \end{array}$$

Simplifying the Hessian matrix, we have


$$|H|=\frac{\nu -s}{b-a}[\frac{\partial^{2}\varphi (\tau_{N})}{\partial \tau_{N}^{2}}\cdot \bar{\mu }(a-Q_{N}+\lambda \varphi (\tau_{N})\bar{\mu })+k\frac{\nu -s}{b-a}$$

According to the assumption $\frac{\partial^{2}\varphi (\tau_{N})}{\partial \tau_{N}^{2}}<0$ and $(a-Q_{N}+\lambda \varphi (\tau_{N})\bar{\mu })<0$, we can prove that |H|>0. Owing to the first-order minor—specifically, the negative definiteness of the Hessian matrix—a unique equilibrium solution is guaranteed to exist.

By setting $\frac{\partial \Pi_{N}}{\partial Q_{N}}$ and $\frac{\partial \Pi_{N}}{\partial \tau_{N}}$ to zero, we have


$$F(Q_{N}-\lambda \varphi (\tau_{N})\bar{\mu })=\frac{\nu -c}{\nu -s}$$ (A1)

$$(\nu -c)\lambda \bar{\mu }\left(\frac{\partial \varphi (\tau_{N})}{\partial \tau_{N}}\right)-k\tau_{N}=0$$ (A2)

Substituting $\varphi (\tau_{N})=\eta \sqrt{\tau_{N}}$ into Eq (A2), we obtain $\tau_{N}^{*}={\left[\frac{\lambda \eta \bar{\mu }(\nu -c)}{2k}\right]}^{3/2}.$

We derive that the maximum preservation effort is $\eta^{-2}$ due to φ(τ)≤1. Hence, $\tau_{N}^{*}=\min\left\{\eta^{-2},{\left[\frac{\lambda \eta \bar{\mu }(\nu -c)}{2k}\right]}^{3/2}\right\}.$ Substituting $\tau_{N}^{*}$ into Eq (A1), the conclusion of Formula (5) is obtained.

***Proof of Theorem 2.*** By substituting Eq (A1) into $p^{*}=r^{*}=\nu -\xi (\beta \nu -s)=\nu F(Q^{*}-\lambda \varphi (\tau )\bar{\mu })(\beta \nu -s)$, we obtain Formula (A3):


$$\nu =p^{*}+\frac{p^{*}-c}{p^{*}-s}(\beta \nu -s).)$$ (A3)

Simplifying Formula (A3), we derive the expression of $p^{*}$ as follows:


$$p^{*}=s+\frac{(1-\beta )\nu }{2}+\sqrt{\frac{(1-\beta {)}^{2}\nu^{2}}{4}+\beta \nu (c-s)+s^{2}-cs}.$$ (A4)

Substituting β=s/ν and β=1 into Formula (A4), Formula (7) is obtained.

***Proof of Corollary 1.*** As per Formula (A4), holding the parameters *ν*, *c*, and *s* constant, a reduction in *β* requires an increase in $p^{*}$. Clearly, $p^{*}$ is a decreasing function with respect to *β*.

***Proof of Theorem 3.*** The proof follows a similar approach to that of Theorem 1.

***Proof of Corollary 2.*** Deriving the first-order partial derivatives of Eqs (8) and (9) with respect to *η* and *k*, respectively, readily provides the necessary conclusions.

***Proof of Corollary 3.*** Rewriting Eq (8), the optimal preservation effort is given by


$$\begin{array}{c} \tau_{S}^{*}=\left\{ \begin{array}{cc} \eta^{-2}, & \text{if }\bar{\mu }\geq \mu_{S},\\ {\left[\frac{\lambda \eta \bar{\mu }(p^{*}-c)}{2k}\right]}^{3/2}, & \text{if }\bar{\mu }<\mu_{S}. \end{array}\right.\; \end{array}$$

It is obvious that $\tau_{S}^{*}$ is a non-increasing function with respect to *β*, because $p^{*}$ is a decreasing function with respect to *β*, as shown in Corollary 2. Similarly, according to Formula (9), $Q_{S}^{*}$ is a decreasing function with respect to *β*.

***Proof of Corollary 4.*** Comparing Formulas (4) and (8), it is evident that $\tau_{S}^{*}<\tau_{N}^{*}$, given that $p^{*}<\nu$. Setting the threshold values for $\bar{\mu }$ to be $\mu_{N}$ and $\mu_{S}$ if $\tau_{N}^{*}=\eta^{-2}$ and $\tau_{S}^{*}=\eta^{-2}$, we obtain two threshold values as follows:


$$\mu_{N}=2\lambda^{-1}\eta^{-7/3}k(\nu -c{)}^{-1},\;\mu_{S}=2\lambda^{-1}\eta^{-7/3}k(p^{*}-c{)}^{-1}.$$

Deriving the first-order partial derivatives of $\tau_{N}^{*}$ and $\tau_{S}^{*}$ with respect to $\bar{\mu }$, we obtain


$$\frac{\partial \tau_{S}^{*}}{\partial \bar{\mu }}=1.5\;(\bar{\mu }{)}^{1/2}{\left(\frac{\lambda \eta (p^{*}-c)}{2k}\right)}^{3/2},\;\frac{\partial \tau_{N}^{*}}{\partial \bar{\mu }}=1.5\;(\bar{\mu }{)}^{1/2}{\left(\frac{\lambda \eta (\nu -c)}{2k}\right)}^{3/2}.$$

When $\bar{\mu }<\mu_{N}$ and $p^{*}<\nu$, it follows that $\frac{\partial \tau_{S}^{*}}{\partial \bar{\mu }}$ is less than $\frac{\partial \tau_{N}^{*}}{\partial \bar{\mu }}$.

When $\mu_{N}\leq \bar{\mu }<\mu_{S}$ and $\tau_{N}^{*}=\eta^{-2}$, it follows that $\frac{\partial \tau_{S}^{*}}{\partial \bar{\mu }}>0$ and $\frac{\partial \tau_{N}^{*}}{\partial \bar{\mu }}=0$.

When $\bar{\mu }\geq \mu_{S}$ and $\tau_{N}^{*}=\tau_{S}^{*}=\eta^{-2}$, it follows that $\frac{\partial \tau_{N}^{*}}{\partial \bar{\mu }}=\frac{\partial \tau_{S}^{*}}{\partial \bar{\mu }}=0.$

***Proof of Corollary 5***

(i) Comparing Formulas (4) and (8), it is obvious that $Q_{N}^{*}>Q_{S}^{*}$, due to $\nu >p^{*}$.
(ii) Rewriting Formulas (5) and (9) yields Formula (A5) and (A6).

$$\begin{array}{c} Q_{N}^{*}=\left\{ \begin{array}{cc} (\lambda \eta \bar{\mu }{)}^{7/4}{\left(\frac{\nu -c}{2k}\right)}^{3/4}+y_{N}, & \text{if }\bar{\mu }<\mu_{N},\\ \lambda \bar{\mu }+y_{N}, & \text{if }\bar{\mu }\geq \mu_{N}. \end{array}\right.\; \end{array}$$ (A5)

$$\begin{array}{c} Q_{S}^{*}=\left\{ \begin{array}{cc} (\lambda \eta \bar{\mu }{)}^{7/4}{\left(\frac{p^{*}-c}{2k}\right)}^{3/4}+y_{S}, & \text{if }\bar{\mu }<\mu_{S},\\ \lambda \bar{\mu }+y_{S}, & \text{if }\bar{\mu }\geq \mu_{S}. \end{array}\right.\; \end{array}$$ (A6)

Using Formulas (A5) and (A6), we reformulate the expressions for $Q_{S}^{*}$ and $Q_{N}^{*}$ under varying market sizes as follows:

When $\bar{\mu }<\mu_{N}$,


$$Q_{N}^{*}=(\lambda \eta \bar{\mu }{)}^{7/4}{\left(\frac{\nu -c}{2k}\right)}^{3/4}+y_{N},$$ (A7-1)

$$Q_{S}^{*}=(\lambda \eta \bar{\mu }{)}^{7/4}{\left(\frac{p^{*}-c}{2k}\right)}^{3/4}+y_{S}.$$ (A7-2)

When $\mu_{N}\leq \bar{\mu }<\mu_{S}$,


$$Q_{N}^{*}=\lambda \bar{\mu }+y_{N},$$ (A8-1)

$$Q_{S}^{*}=(\lambda \eta \bar{\mu }{)}^{7/4}{\left(\frac{p^{*}-c}{2k}\right)}^{3/4}+y_{S}.$$ (A8-2)

When $\bar{\mu }\geq \mu_{S}$,


$$Q_{N}^{*}=\lambda \bar{\mu }+y_{N},$$ (A9-1)

$$Q_{S}^{*}=\lambda \bar{\mu }+y_{S}.$$ (A9-2)

Deriving the first-order partial derivatives of Formulas (A7-1) and (A7-2) with respect to $\bar{\mu }$, respectively, we obtain


$$\frac{\partial Q_{N}^{*}}{\partial \bar{\mu }}=\frac{7}{4}(\lambda \eta {)}^{7/4}(\bar{\mu }{)}^{3/4}{\left(\frac{\nu -c}{2k}\right)}^{3/4},$$

$$\frac{\partial Q_{S}^{*}}{\partial \bar{\mu }}=\frac{7}{4}(\lambda \eta {)}^{7/4}(\bar{\mu }{)}^{3/4}{\left(\frac{p^{*}-c}{2k}\right)}^{3/4}.$$

It is obvious that $\frac{\partial Q_{S}^{*}}{\partial \bar{\mu }}<\frac{\partial Q_{N}^{*}}{\partial \bar{\mu }}$, because $p^{*}<\nu .$

**(ii)** Deriving the first-order partial derivatives of Formulas (A8-1) and (A8-2) with respect to $\bar{\mu }$, respectively, we obtain


$$\frac{\partial Q_{N}^{*}}{\partial \bar{\mu }}=\lambda ,\;\frac{\partial Q_{S}^{*}}{\partial \bar{\mu }}=\frac{7}{4}(\lambda \eta {)}^{7/4}(\bar{\mu }{)}^{3/4}{\left(\frac{p^{*}-c}{2k}\right)}^{3/4}.$$

To ensure that the hypothesis $\frac{\partial Q_{S}^{*}}{\partial \bar{\mu }}<\frac{\partial Q_{N}^{*}}{\partial \bar{\mu }}$ holds, the retail price must satisfy the condition that $p^{*}<{\left(\frac{4}{7}\right)}^{3/4}(\nu -c)+c.$

**(iii)** Deriving the first-order partial derivatives of Formulas (A9-1) and (A9-2) with respect to $\bar{\mu }$, respectively, we obtain $\frac{\partial Q_{N}^{*}}{\partial \bar{\mu }}=\frac{\partial Q_{S}^{*}}{\partial \bar{\mu }}=\lambda .$
